# Complete mitochondrial genome of *Goniurosaurus zhoui* (Squamata: Sauria: Eublepharidae)

**DOI:** 10.1080/23802359.2019.1573116

**Published:** 2019-10-04

**Authors:** Jichao Wang, Yifan Chen, Xiaofei Zhai, Xingyu Tao, Tongliang Wang

**Affiliations:** Ministry of Education Key Laboratory for Ecology of Tropical Islands, College of Life Sciences, Hainan Normal University, Haikou, China

**Keywords:** *Goniurosaurus*, mitogenome, phylogeny

## Abstract

In this study, using Illumina sequencing data, we assembled the complete mitogenome of *Goniurosaurus zhoui*, which consists of 16,803 base pairs, comprising 13 protein-coding genes, 2 ribosomal RNA genes, 21 tRNA genes, and non-coding regions (D-loop), and has an overall A + T content of 60.39%. Using three complete 16S rRNA gene and 13 partial sequence data of the 16S rRNA gene, including that of *G. zhoui,* we reconstructed the phylogeny of the genus *Goniurosaurus*. The result of the Bayesian inference tree revealed four major, distinctly separated groups.

*Goniurosaurus zhoui* is a newly identified species in the genus *Goniurosaurus*. It was described based on a quantitative comparison of morphological traits and egg size in 2018 on Hainan Island (Zhou et al. 2018). It is endemic to Hainan Island, similar to the other two *Goniurosaurus* species, *G. bawanglingensis,* and *G*. *hainanensis* (Grismer et al. 1999, 2002; Blair et al. 2009). In the previous classification*, Goniurosaurus* species were separated into four major groups based on morphological and molecular analyses (Wang et al. 2014). Previous molecular analyses based on mitochondrial or nuclear DNA did not include *G. bawanglingensis* and *G. zhoui*, so that the relationships among three endemic *Goniurosaurus* species to Hainan Island are still unclear. In the study of Liang et al. (2018) the *G. luii* group with high support, but it does not include *G. bawanglingensis*, all species on Hainan Island formed a monophyletic group.

In this study, we determined the complete mitochondrial genome of *G. zhoui* for the first time. The specimen (voucher number: HNNU2017072601) was collected from the central area of Hainan Island (we have not provided the exact location of the collection in order to protect the species). DNA was extracted from fresh tail tissue using the standard phenol-chloroform method (Köchl et al. 2005). The complete mitogenome sequence of *G. zhoui* was determined using high-throughput sequencing technology and using the *G. luii* mitogenome (GenBank accession number: NC026105.1) as a reference.

The complete mitogenome of *G. zhoui* is 16,803 bp in length with a 60.39% A + T content. The genome contains the typical components, including 13 protein-coding genes, 2 ribosomal RNA genes, 21 tRNA genes, and non-coding regions (D-loop). The arrangement of the protein-coding and ribosomal RNA genes is similar to that found in typical vertebrates. However, there was a difference in the arrangement of tRNA genes, because of the deletion of the tRNA-Pro gene.

Three complete 16S rRNA genes, including those of *G. zhoui, G. bawanglingensis, and G. luii*, and 13 partial sequences of the 16S rRNA gene were used to reconstruct the phylogeny of, using Bayesian inference (BI) methods implemented with MrBayes (version 3.2.2); *Gekko gecko* 16S rRNA gene was used as the outgroup. The result of the BI tree is similar to Liang et al. (2018) (Figure 1). Our results also suggest that species on Hainan Island formed a monophyletic group. The classification and molecular analyses of species relationships within the *Goniurosaurus* genus still needed more molecular data, such as the mitochondrial sequence of *G. kadoorieorum* and *G. kwangsiensis*.

**Figure 1.**
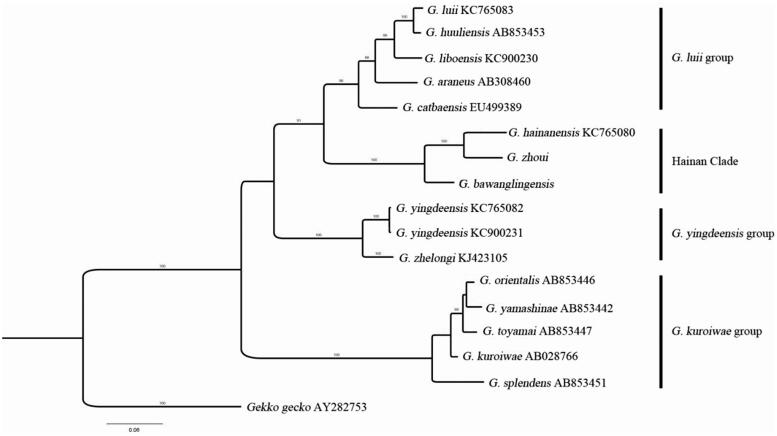
A Bayesian inference tree based on partial nucleotide sequence data of the 16S rRNA gene of 15 *Goniurosaurus* species.
